# The Effect of Silibinin on Protein Expression Profile in White Adipose Tissue of Obese Mice

**DOI:** 10.3389/fphar.2020.00055

**Published:** 2020-02-28

**Authors:** Fei Wang, Shuchun Chen, Luping Ren, Yichao Wang, Zelin Li, Tiantian Song, He Zhang, Qiwen Yang

**Affiliations:** ^1^ Graduate School of Hebei Medical University, Shijiazhuang, China; ^2^ Department of Endocrinology, Hebei General Hospital, Shijiazhuang, China; ^3^ North China University of Science and Technology, Tangshan, China; ^4^ Hebei North University, Zhangjiakou, China

**Keywords:** silibinin, white adipose tissue, protein expression profile, liquid chromatography-tandem mass spectrometry, Tandem Mass Tag

## Abstract

**Objective:**

To investigate the effect of silibinin on the protein expression profile of white adipose tissue (WAT) in obese mice by using Tandem Mass Tag (TMT) and liquid chromatography-tandem mass spectrometry (LC-MS/MS).

**Methods:**

According to experimental requirements, 36 C57BL/6JC mice were randomly divided into normal diet group (WC group), high fat diet group (WF group), and high fat diet + silibinin group (WS group). WS group was intragastrically administered with 54 mg/kg body weight of silibinin, and the WC group and the WF group were intragastrically administered with equal volume of normal saline. Serum samples were collected to detect fasting blood glucose and blood lipids. IPGTT was used to measure the blood glucose value at each time point and calculate the area under the glucose curve. TMT combined with LC-MS/MS were used to study the expression of WAT, and its cellular processes, biological processes, corresponding molecular functions, and related network molecular mechanisms were analyzed by bioinformatics. Finally, RT-PCR and LC-MS/MS were used to detect the mRNA and protein expressions of FABP5, Plin4, GPD1, and AGPAT2, respectively.

**Results:**

Although silibinin did not reduce the mice's weight, it did improve glucose metabolism. In addition, silibinin decreased the concentration of TC, TG, and LDL-C and increased the concentration of HDL-C in the serum of mice. In the WF/WS group, 182 differentially expressed proteins were up-regulated and 159 were down-regulated. While in the WS/WF group, 362 differentially expressed proteins were up-regulated and 176 were down-regulated. Further analysis found that these differential proteins are mainly distributed in the peroxisome proliferation-activated receptor (PPAR), lipolysis of fat cells, metabolism of glycerides, oxidative phosphorylation, and other signaling pathways, and participate in cell processes and lipid metabolism through catalysis and integration functions. Specifically, silibinin reduced the expression of several key factors such as FABP5, Plin4, GPD1, and AGPTA2.

**Conclusion:**

High fat diet (HFD) can increase the expression of lipid synthesis and transport-related proteins and reduce mitochondrial related proteins, thereby increasing lipid synthesis, reducing energy consumption, and improving lipid metabolism *in vivo*. Silibinin can reduce lipid synthesis, increase energy consumption, and improve lipid metabolism in mice *in vivo*.

## Introduction

Obesity is a chronic metabolic disease caused by heredity, lifestyle, environment, and other factors, and the imbalance between energy intake and consumption was the root cause. With the increasing incidence of obesity, the incidence of diabetes, hyperlipidemia, fatty liver, cardiovascular disease, atherosclerosis, and other diseases is also increasing (Wild et al., 2004). It is estimated that by 2030, there will be 366 million people suffering from diabetes globally, most of whom are caused by obesity (Zimmet et al., 2001).

Adipose tissue is the main site for energy storage and it is found throughout the body in distinct subcutaneous and visceral depots, including white adipose tissue (WAT) and brown adipose tissue (BAT) (van Dam et al., 2017). Among them, WAT is mainly used to store energy, while BAT is mainly used to convert excess energy into heat energy. Adipose tissue is also the important endocrine organ of the human body, which can secrete a variety of adipocytes that participate in the differentiation, metabolism, oxidative stress, inflammation, and apoptosis of fat cells (Crewe et al., 2017). Excess energy is stored in fat cells in the form of TG and promotes the increase in size and number of fat cells and reduces lipid toxicity. However, it will become dysfunctional when WAT storage exceeds its threshold (Brestoff and Artis, 2015). As a result, lipids will be deposited in the liver, pancreas, and other important organs, leading to chronic inflammation and occurrence of diabetes, insulin resistance, cardiovascular disease, obesity, and other diseases, which can further aggravate adipose tissue function (Ibarretxe et al., 2016). Therefore, it is of great significance to intervene in lipid synthesis and reduce fat inflammation to prevent the occurrence of obesity-related complications (Hubler and Kennedy, 2016).

Silymarin, a flavonoid complex isolated from the seeds of milk thistle, is composed primarily of silibinin with small amounts of other stereoisomers (Salomone et al., 2016). As a classic liver-protecting drug, silibinin is widely used in the treatment of fatty liver, hepatitis, cirrhosis, and other liver diseases, and has achieved a significant effect. A previous study indicated that silibinin can reduce liver lipid deposition by improving glycolipid metabolism, insulin resistance, and oxidative stress in the liver (Liu et al., 2019). In addition, it was also found that silibinin also has lipid-lowering, anti-inflammatory, anti-oxidative stress, anti-atherosclerosis, cognitive function improvement, and anti-obesity effects (Tajmohammadi et al., 2018). Recently, in the research progress of silibinin, a new study has found that silibinin can also increase the bilirubin level in plasma to play an anti-oxidative stress and inhibit the effect of lipid oxidation (Suk et al., 2019). However, few studies have been done on the mechanism of silibinin in visceral adipose tissue.

In this study, we used TMT combined with LC-MS/MS to observe the changes of WAT protein in epididymis of mice with high-fat diet before and after silibinin intervention and further explored the mechanism of silibinin on adipose tissue function in obese people, which provides a new basis for the prevention and treatment of obesity and its complications.

## Materials and Methods

### Animals and Experimental Groups

Male C57BL/6JC mice (7 weeks old) used in this study were purchased from Beijing Weilitonglihua Laboratory Animal Technology Co. Ltd. They were housed in a pathogen-free environment (22 ± 2°C, 55 ± 10% humidity, and 12—12 h/light–dark cycle) with free access to a standard laboratory diet and water. The animal experimental procedures were approved by the Animal Ethics Committee of Hebei People's Hospital. All experiments were carried out in accordance with the National Institute of Health Guide for the Care and Use of Laboratory Animals.

After one week of adaptive feeding, the animals were randomly divided into normal diet group (WC group, n = 12) and high fat diet group (WF group, n = 24). In the WC group, mice were fed with normal diet consisting of 70% carbohydrate, 10% fat, and 20% protein. The total calories were 348 kcal/100g. In the WF group, mice were fed with high fat diet consisting of 20% carbohydrate, 60% fat, and 20% protein. The total calories were 524 kcal/100g. Mice in each group were fed daily with equal calories and fed with water freely. The food intake was recorded daily. Fasting weight was measured every week and changes were recorded. After 4 weeks, the mice in the WF group were further subdivided into two groups: WF group (n = 12) and WF + silibinin group (WS group, n = 12). The WS group was intragastrically administered with 54 mg/kg body weight of silibinin, and the WC group and the WF group were intragastrically administered with equal volume of normal saline for 4 weeks. After 4 weeks of drug intervention, blood glucose values at each time point were measured by IPGTT, and AUCglu was calculated.

### Intraperitoneal Glucose Tolerance Test (IPGTT)

After fasting for 12 hours, FPG was measured by fasting blood drops from the tail tip on the Roche rapid glucose meter strip, followed by intraperitoneal injection of 50% glucose 2 g/kg. Blood glucose values of the tail tip were measured at 15, 30, 60, and 120 minutes after glucose injection, and the area under the glucose curve of each mouse was calculated. The calculation formula is: AUCglu = (0′+15′)/8+(15′+30′)/8+(30′+60′)/4+(60′+120)/2.

### Sample Collection and Preparation

All mice fasted overnight. After weighing, the mice were anesthetized intraperitoneally with 1% pentobarbital sodium (60 mg/kg), and blood was taken from the eyeball. The blood was then coagulated for 30 min at 4°C and centrifuged at 3,000 × g for 20 min. The serum supernatant was collected and stored at –80°C. After taking blood from the eyeball, the adipose tissue of the epididymis was removed by laparotomy and quickly placed in liquid nitrogen, followed by cryopreservation at –80°C.

### Detection of Mouse Serum Indicators

Insulin levels in mice were determined by antibody sandwich ELISA; the insulin ELISA kit was purchased from ALPCO, USA. Blood samples were transferred to the tubes containing anticoagulants (4.80 g/L citric acid, 14.70 g/L glucose, and 13.20 g/L tri-sodium citrate). Measurements of serum TC, TG, HDL-C, and LDL-C levels in the samples were performed by enzymatic methods with commercially available kits (RANDOX Laboratories Ltd., Ardmore, Diamond Road, CrumlinCo. Antrim, United Kingdom, BT29 4QY; Daiichi Pure Chemicals Co., Ltd., Tokyo, Japan). All determinations were performed with full automatic blood biochemical analyzer (Sysmex Shanghai Ltd., Shanghai, China).

### Protein Digestion and Peptide Labelling

The epididymal adipose tissue of mice was grinded with liquid nitrogen, added in lysate (8M urea, 1% protease inhibitor, and 2 mm EDTA), and ultrasonicated 10 times for 10 seconds once, followed by centrifugation at 1200 g at 4°C for 10 minutes. The supernatant was added with a final concentration of 5 mmol/LDTT and reduced at 56°C for 30 min. LAA with a final concentration of 11 mmol/L was added and incubated at room temperature in the dark for 15 min. Samples were then digested using 40 μL of 0.05 g/L Trypsin (Bruker, Beijing, China) at 37°C for 14 to 16 hours. After trypsin digestion, peptide was desalted by Strata X C18 SPE column (Phenomenex) and vacuum-dried. Peptide was reconstituted in 0.5 M TEAB and processed according to the manufacturer's protocol for TMT kit/iTRAQ kit. Briefly, one unit of TMT/iTRAQ reagent were thawed and reconstituted in acetonitrile. The peptide mixtures were then incubated for 2 hours at room temperature and pooled, desalted, and dried by vacuum centrifugation.

### Lc‐Ms/Ms Analysis

The desalted peptides were dissolved in a buffer containing 2% acetonitrile 0.1% formic acid before separation by high pH on Q ExactiveTM HF-X using a Zorbax C18 column (2.1 × 150 mm). Peptides were eluted with a linear gradient of 20 mM ammonium formate, 2% ACN to 20 mM ammonium formate, 90% ACN at 0.2 mL/min. The 95 fractions were concatenated into 12 fractions and dried down. Each fraction was analyzed by electrospray ionization mass spectrometry using the Shimadzu Prominence nano HPLC system [Shimadzu] coupled to a 5600 TripleTOF mass spectrometer [Sciex]. Samples were loaded onto an Agilent Zorbax 300SB-C18, 3.5 μm [Agilent Technologies] and separated with a linear gradient of water/acetonitrile/0.1% formic acid (v/v). Fourteen percent of the labeled sample was loaded on the mass spectrometer.

### Database Searching

The tandem mass spectra were extracted and analyzed for the removal of isotopes and resolved by the Mascot Distiller software suite from Matrix Science, Boston, MA (version 2.6). Mass spectral data was retrieved using Maxquant (v1.5.2.8). At the same time, all mass spectrometry results were evaluated using a reverse database search method to estimate the false positive rate (FDR) of data caused by random matches. The FDR of proteins and peptides is <1%.

### Bioinformatic Analysis

Gene Ontology (GO) is a comprehensive resource of computable knowledge regarding the functions of genes and gene products. Kyoto Encyclopedia of Genes and Genomes (KEGG) is a tool for mapping differentially expressed genes to increase our knowledge of the molecular interaction and reaction networks. The InterPro database can classify protein sequences into families, predicting their domains and important sites. Here, GO and InterProScan software were used to preliminarily analyze the cellular processes of the differential proteins, the biological processes involved, and the corresponding molecular functions. InterPro database and InterProScan software were used to annotate the protein domain of the identified protein. KEGG was used to annotate protein pathways and classify these pathways according to the KEGG website pathway hierarchy classification method.

### Real-Time PCR Analysis

Total RNA was harvested and extracted using TRIzol kit (QIAGEN, Hilden, Germany) according to the manufacturer's protocol, and then it was reverse transcribed to cDNA using a Sensiscript RT kit (ThermoFisher Scientific Inc., USA). Subsequently, RT-qPCR was performed. The thermocycling conditions were as follows: 95°C for 5 min; 30 cycles of 95°C for 30 sec, 56°C for 30 sec, and extension at 72°C for 1 min. Relative quantities of mRNA were calculated using the 2^-∆∆Ct^ method and normalized to housekeeping gene β-actin. PCR primer sequences are shown below: β-actin F: GGCTGTATTCCCCTCCATCG, β-actin R: CCAGTTGGTAACAATGCCATGT; Fabp5 F: ATGGCAACAACATCACGG, Fabp5 R: TCATCAAACTTCTCTCCCAGG. GPD1 F: TGGAGAAGGAGATGCTAAATGG, GPD1 R: TGTGTTGGAGAATGCTGTGC. AGPAT2 F: GTTCGTTCGGTCCTTCAAG, AGPAT2 R: CCTCCAGTTTCTTCTGTCCG. Plin4 F: GCAGTATCTGGAGGTGTGATG, Plin4 R: TGTGTCCTTCGTATTGGTGAG.

### Proteomics Validation

The tryptic peptides were dissolved in 0.1% formic acid (solvent A), directly loaded onto a home-made reversed-phase analytical column. The gradient was comprised of an increase from 6% to 23% solvent B (0.1% formic acid in 98% acetonitrile) over 38 min, 23% to 35% in 14 min and climbing to 80% in 4 min, then holding at 80% for the last 4 min, all at a constant flow rate of 700 nL/min on an EASY-nLC 1000 UPLC system. The peptides were subjected to NSI source followed by tandem mass spectrometry (MS/MS) in Q Exactive™ Plus (Thermo) coupled online to the UPLC.

### Statistical Analysis

All statistical analyses were performed using the GraphPad Prism 5.0 software (San Diego, CA). The experimental data are presented as mean ± SD. The multiple comparisons were analyzed using one-way ANOVA followed by Bonferroni post test. χ^2^ text was used to test for differentially expressed proteins. A P < 0.05 was defined as statistically significant.

## Results

### Silibinin Cannot Reduce the Body Weight of Mice

**Figure 1:**
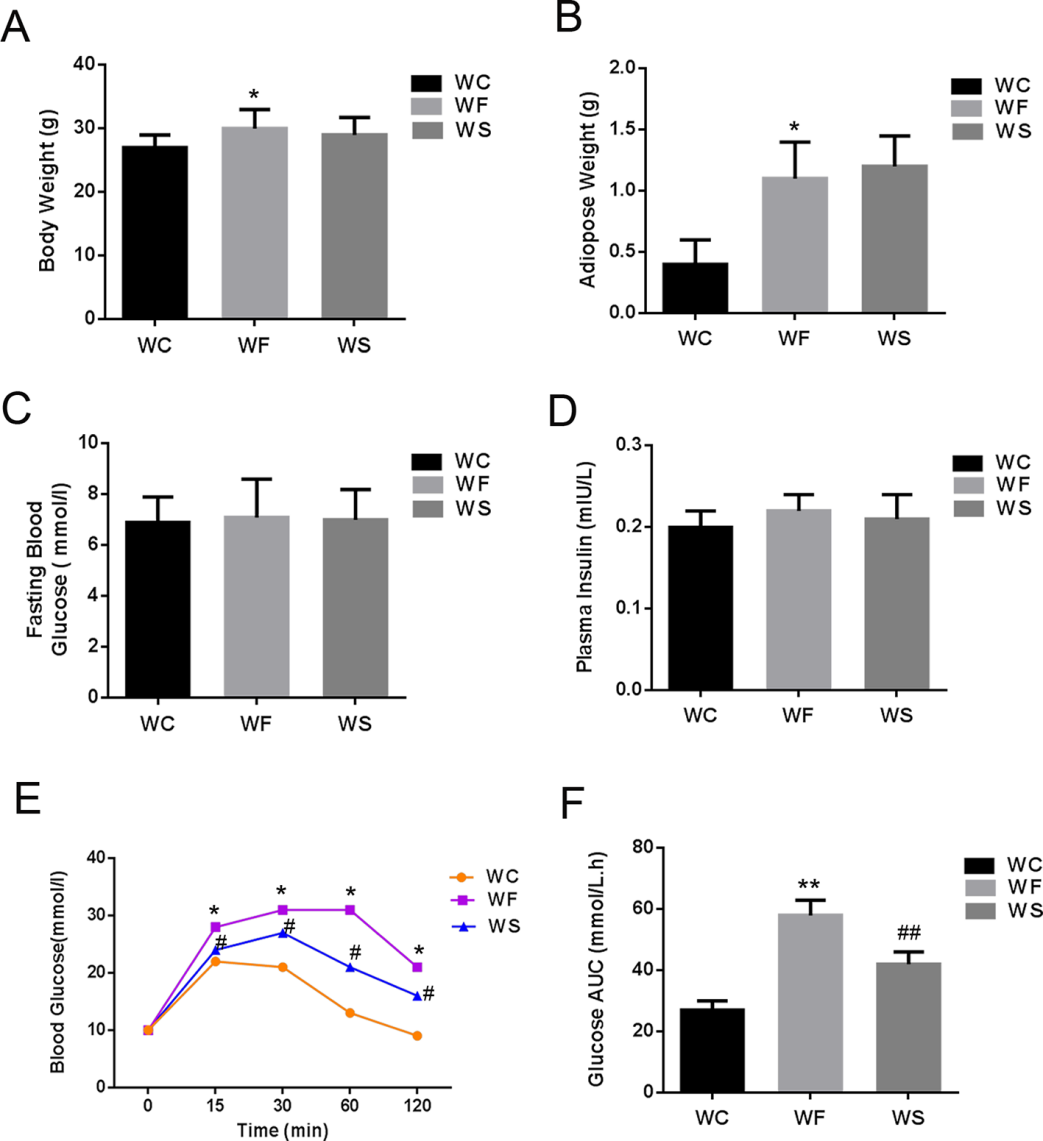
Silibinin cannot reduce the body weight of mice, but can improve glucose metabolism. **(A)** High-fat diet increased the weight of mice in the WF group, while the weight of mice in the WS group was not decreased when silibinin was added; **(B)** High-fat diet increased the adipose weight of mice in the WF group, while the weight of mice in the WS group was not decreased when silibinin was added; **(C)** The comparison of the average fasting blood glucose levels of three groups of mice; **(D)** There was no statistical difference in fasting insulin levels among the three groups; **(E)** The blood glucose of the WF group increased significantly at 15 min, 30 min, 60 min, and 120 min compared with the WC group, and the addition of silibinin significantly reduced its blood glucose concentration; **(F)** The comparison of the glucose AUC of the three groups of mice; *P < 0.05 and **P < 0.01 vs WC; ^#^P < 0.05 and ^##^P < 0.01 vs WF; WC, normal diet; WF, high fat diet; WS, high fat diet + silibinin.

**Figures 1A, B** were a comparison of the average body weight and average adipose tissue weight of three groups of mice. The results showed that the body weight and adipose tissue weight of mice in WF group was higher than that in WC group (*P* < 0.05). The body weight and adipose tissue weight of the WS group was lower than that of the WF group, but there was no statistical difference (*P* > 0.05).

### Silibinin Can Improve Glucose Metabolism

**Figures 1C, D** were a comparison of the average fasting blood glucose levels, fasting insulin and subperitoneal glucose tolerance test of three groups of mice. As presented in **Figures 1C, D**, there was no statistical difference in fasting blood glucose and fasting insulin levels among the three groups (*P* > 0.05). However, the blood glucose of the WF group increased significantly at 15 min, 30 min, 60 min, and 120 min compared with the WC group, and the area under the blood glucose curve increased significantly, with statistically significant differences (*P* < 0.05). More interesting, the addition of silibinin significantly reduced its blood glucose concentration at 15 min, 30 min, 60 min, and 120 min; the area under the blood glucose curve was also significantly reduced, and the differences were statistically significant (**Figures 1E, F**; *P* < 0.05).

**Figure 2:**
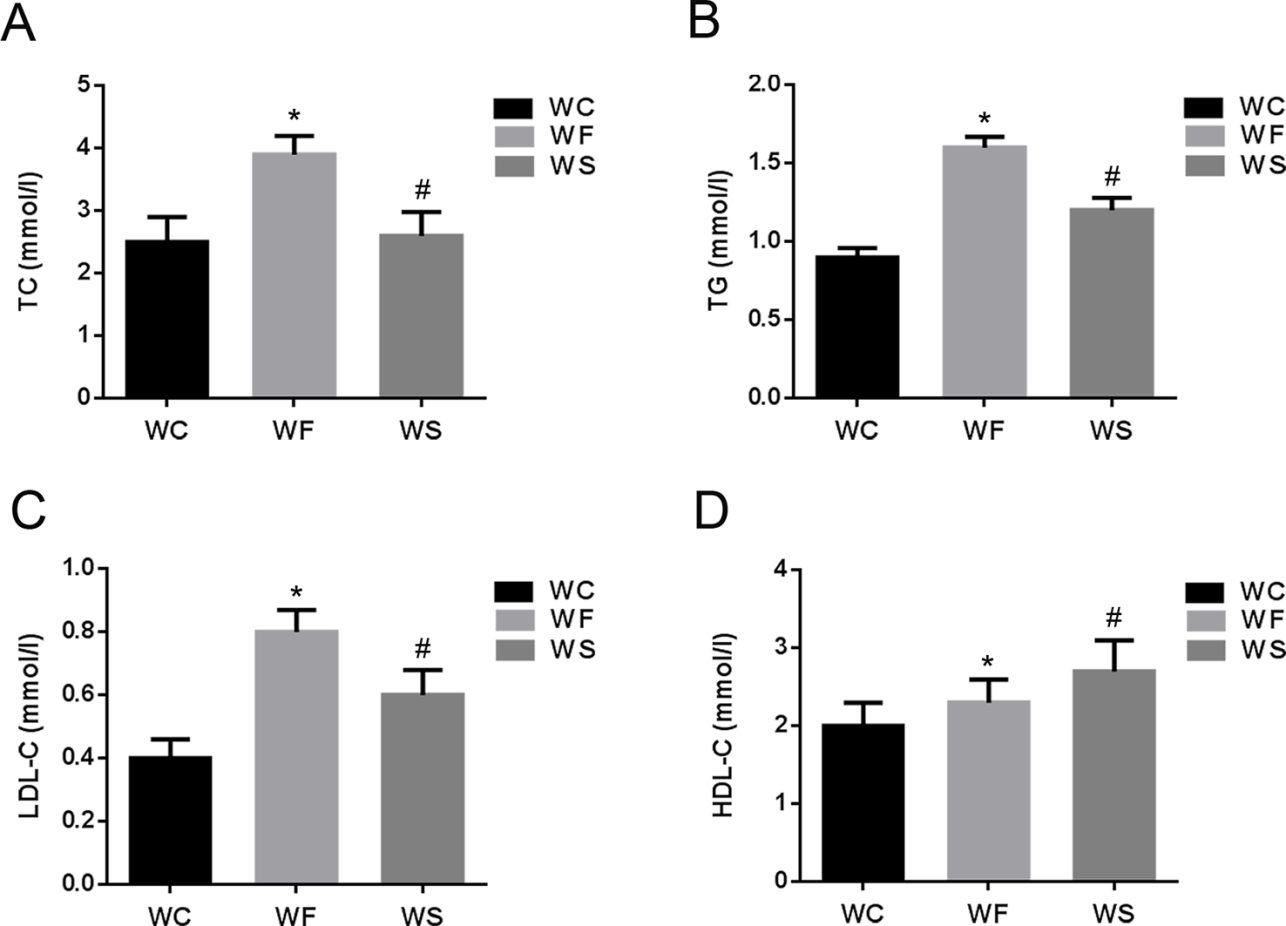
The comparison of TC **(A)**, TG **(B)**, LDL-C **(C)**, and HDL-C **(D)** in mice of groups WC, WF, and WS. *P < 0.05 vs WC, ^#^P < 0.05 vs WF. WC, normal diet; WF, high fat diet; WS, high fat diet + silibinin.

Further, we detected the changes in the concentrations of TC, TG, LDL-C, and HDL-C in the serum. As shown in **Figure 2**, compared with the WC group, the levels of TC, TG, LDL-C, and HDL-C in the serum of the WF group increased significantly; however, when silibinin was added, TC, TG, and LDL-C in the serum of the WS group were significantly reduced, while HDL-C was significantly increased, and the difference was statistically significant (*P* < 0.05).

### Qualitative and Quantitative Analysis of Adipose Tissue Identification Protein

A total of 300,152 secondary spectrograms were obtained by mass spectrometry. After searching the theoretical data of protein, the available number of mass spectrometry secondary spectrogram was 45830, and the utilization rate of spectrogram was 15.27%. Through spectral analysis, we identified a total of 30,121 peptides with a specific peptide of 29,108. In addition, we identified a total of 5108 proteins, of which 4,623 were quantifiable (quantitative proteins indicate at least one comparison group has quantitative information).

### The Identification of Differentially Expressed Proteins

**Figure 3:**
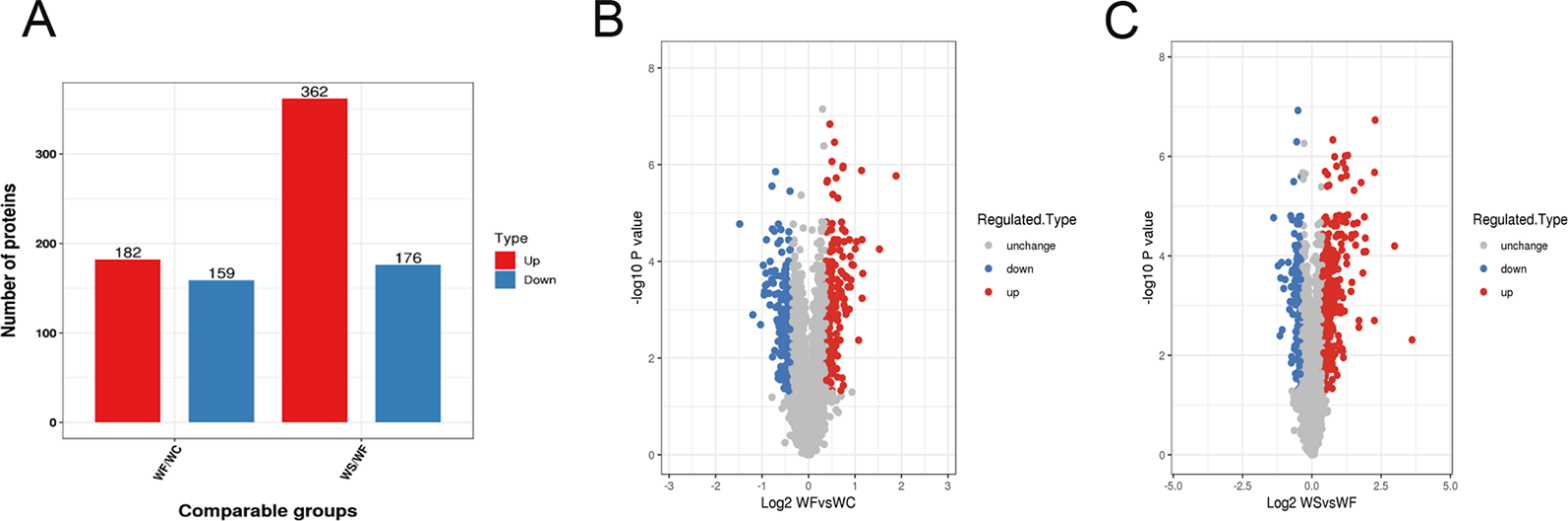
Quantitative information on differential protein identification. **(A)** Histogram of the number distribution of differentially expressed proteins in WF/WC and WS/WF, two different comparison groups; **(B**, **C)** Volcano plot of differentially expressed proteins. The horizontal axis is the relative quantitative value of protein after Log2 logarithm conversion, and the vertical axis is the p-value of difference significance test after Log10 logarithm conversion. The red dots indicate the significantly different expressions of up-regulated proteins, while the blue dots indicate the significantly different expressions of down-regulated proteins.

Mass spectrometry detection of whole protein quantification experiments was repeated three times. When P < 0.05, the threshold of differential expression was more than 1.3 as a significant up-regulation and less than 1.3 as a significant down-regulation. According to the above screening criteria for differentially expressed proteins, we compared the protein expressions of the three treatment groups, and the quantitative information of statistically obtained differentially expressed proteins was shown in **Figure 3A**. In the WF/WC group, there were 341 differentially expressed proteins whose variation multiple was more than 1.3, including 182 up-regulated proteins and 159 down-regulated proteins. In the WS/WF group, there were 538 differentially expressed proteins whose variation multiple was more than 1.3, including 362 up-regulated proteins and 176 down-regulated proteins.

Volcano plot can be very intuitive and reasonable to screen out the differentially expressed genes between the two samples. In order to show the differentially expressed genes more vividly, we drew the volcano plot. The red dots indicated the significantly different expressions of up-regulated proteins, while the blue dots indicated the significantly different expressions of down-regulated proteins (**Figures 3B, C**).

### GO Analysis of Differential Protein

**Figure 4:**
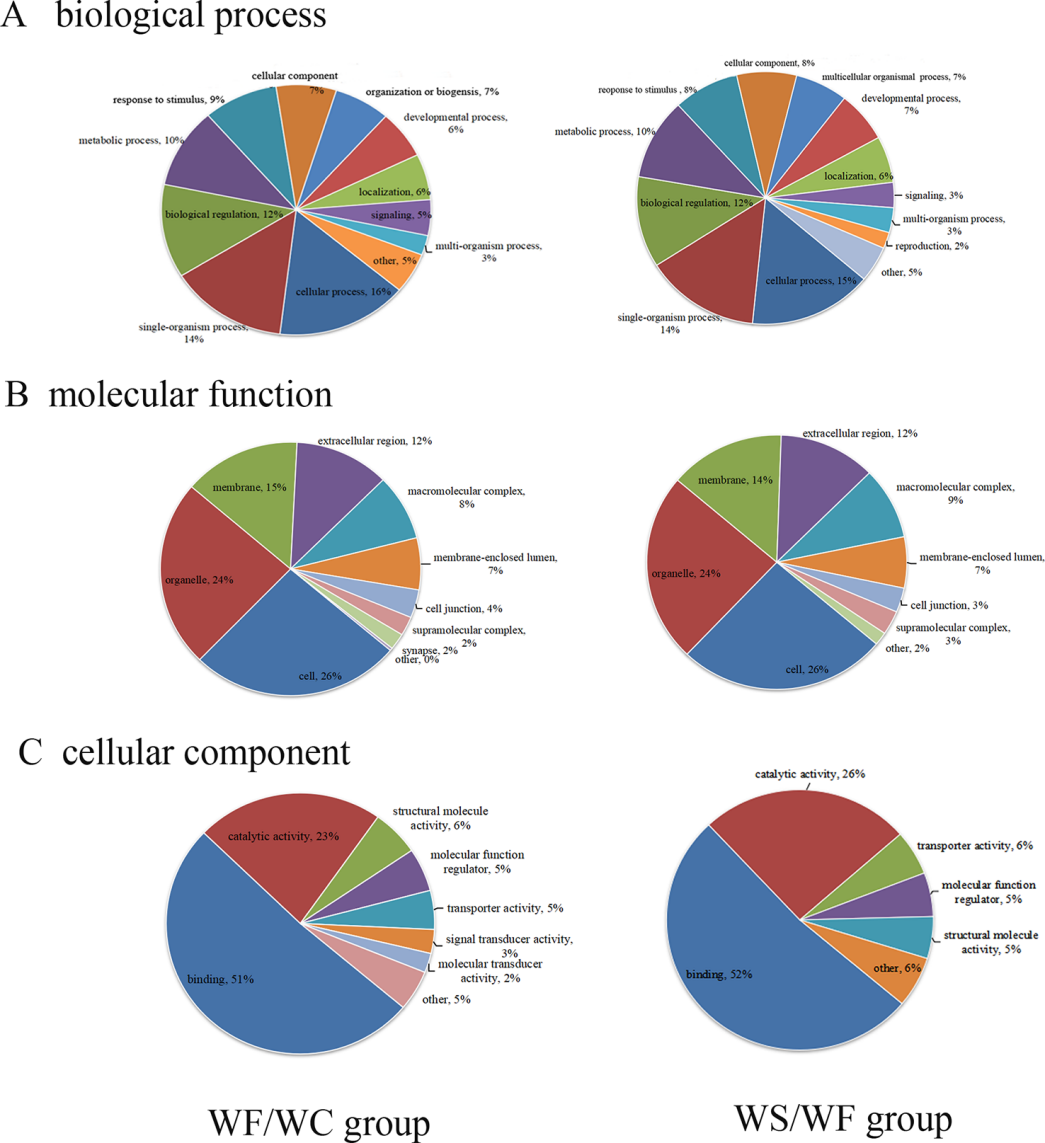
The proportion of differential protein biological process (BP), cell composition (CC), and molecular function (MF) protein species (Left: WF/WC group; Right: WS/WF group). **(A)** The functions of these two groups were basically the same, mainly involved in cell process, single-cell biological process, biological regulation, molecular metabolism, and stress response; **(B)** CC analysis revealed the two groups of differential proteins were mainly involved in the cytoplasmic composition, followed by organelle proteins, cell membrane proteins, extracellular matrices, macromolecular complexes, etc; **(C)** In the MF analysis, the two groups of differential proteins mainly have integration and catalytic functions.

The GO database was used to analyze the final selected differentially expressed proteins and to determine their biological process (BP), molecular function (MF), and cellular component (CC). In the BP analysis, the functions of WF/WC group and WS/WF group were basically the same, mainly involved in cell process, single-cell biological process, biological regulation, molecular metabolism, and stress response (**Figure 4A**). In the CC analysis, we found that the two groups of differential proteins were mainly involved in the cytoplasmic composition, followed by organelle proteins, cell membrane proteins, extracellular matrices, macromolecular complexes, etc (**Figure 4B**). In the MF analysis, the two groups of differential proteins mainly have integration and catalytic functions (**Figure 4C**).

### Subcellular Structure Localization and Classification of Differential Protein

**Figure 5:**
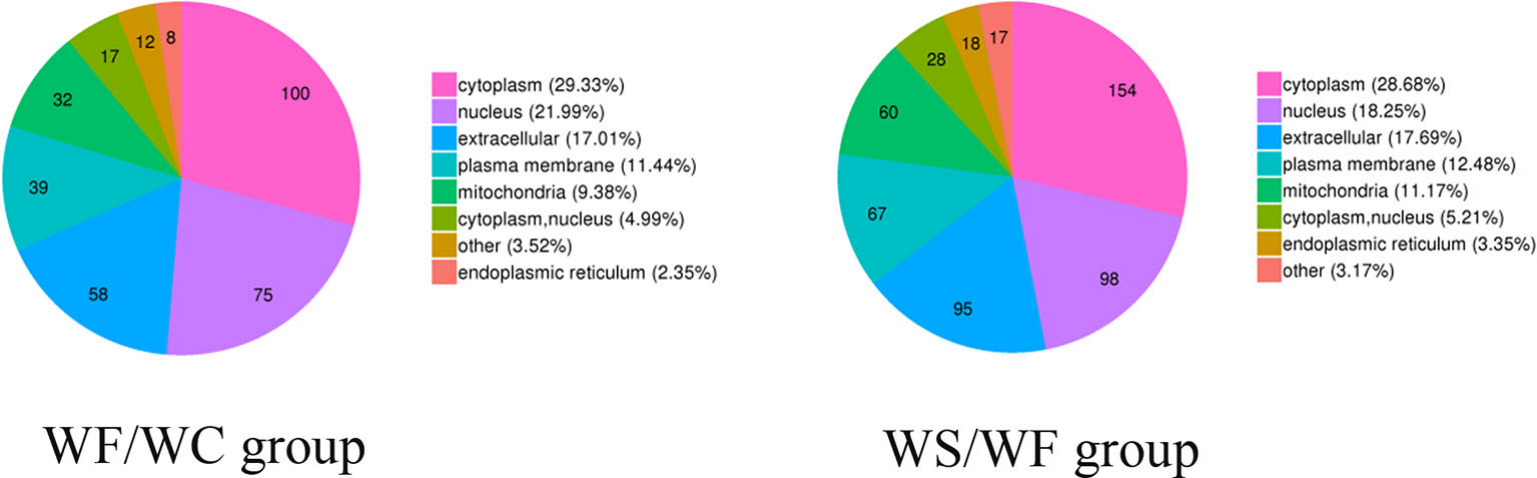
Subcellular localization chart of differentially expressed proteins. The differential proteins in the two comparison groups are roughly distributed, mainly in the cytoplasm, nucleus, extracellular matrix, cell membrane, mitochondria, and endoplasmic reticulum.

The pie chart of **Figure 5** showed the general distribution of the two groups of differential proteins. As can be seen from the figure, the differential proteins in the two comparison groups are roughly distributed, mainly in the cytoplasm, nucleus, extracellular matrix, cell membrane, mitochondria, endoplasmic reticulum and so on.

### Protein Domain Analysis of Differential Protein

**Figure 6:**
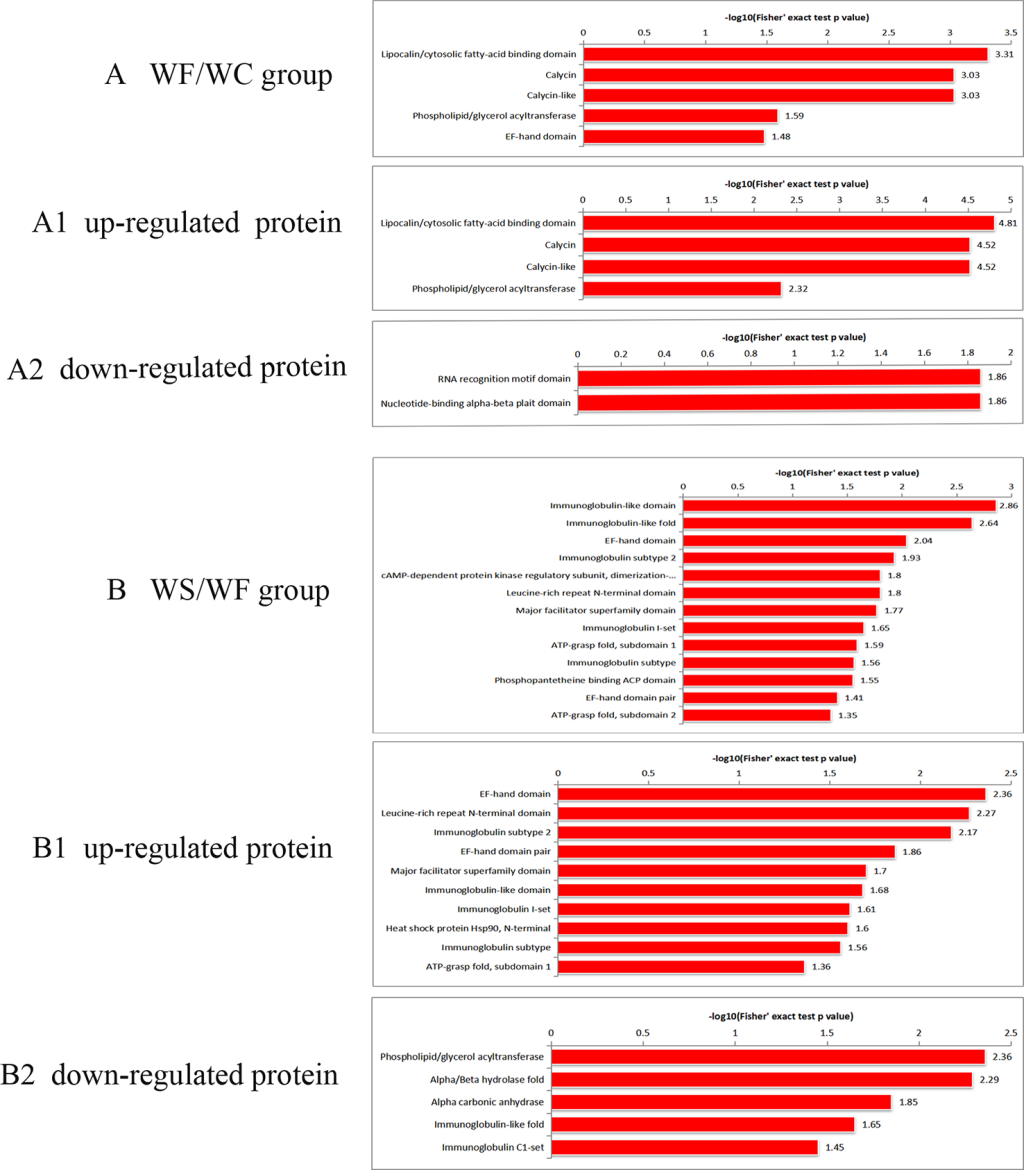
Protein domain enrichment plot of differentially expressed proteins. **(A) **The differential protein domain in WF/WC group; **(A1)** The domain of up-regulated protein in WF/WC group; **(A2)** The domain of down-regulated protein in WF/WC group; **(B)** The differential protein domain in WS/WF group; **(B1)** The domain of up-regulated protein in WS/WF group; **(B2)** The domain of down-regulated protein in WS/WF group. WC, normal diet; WF, high fat diet; WS, high fat diet + silibinin.

It was found that the differential protein domain in the WF/WC group were mainly composed of apolipoprotein/fatty acid binding protein domain, calycin, calycin-like and phospholipid/triglyceride acyltransferase (**Figure 6A**), among which the up-regulated differential protein was concentrated in apolipoprotein/fatty acid binding protein domain (**Figure 6A1**), and the down-regulated protein was concentrated in EF-chiral protein domain (**Figure 6A2**). While the domains of differential proteins in the WS/WF group were mainly concentrated in the immunoglobulin domain, immunoglobulin folding protein, EF-hand domain, immunoglobulin subtype-2 (**Figure 6B**), in which the up-regulated proteins were concentrated in the EF-hand domain and leucine-rich N-terminal domain (**Figure 6B1**), the down-regulated proteins were concentrated in phospholipid/triglyceride acyltransferase, α/β hydrolase (**Figure 6B2**).

### KEGG Pathway Analysis of Differential Protein

**Figure 7:**
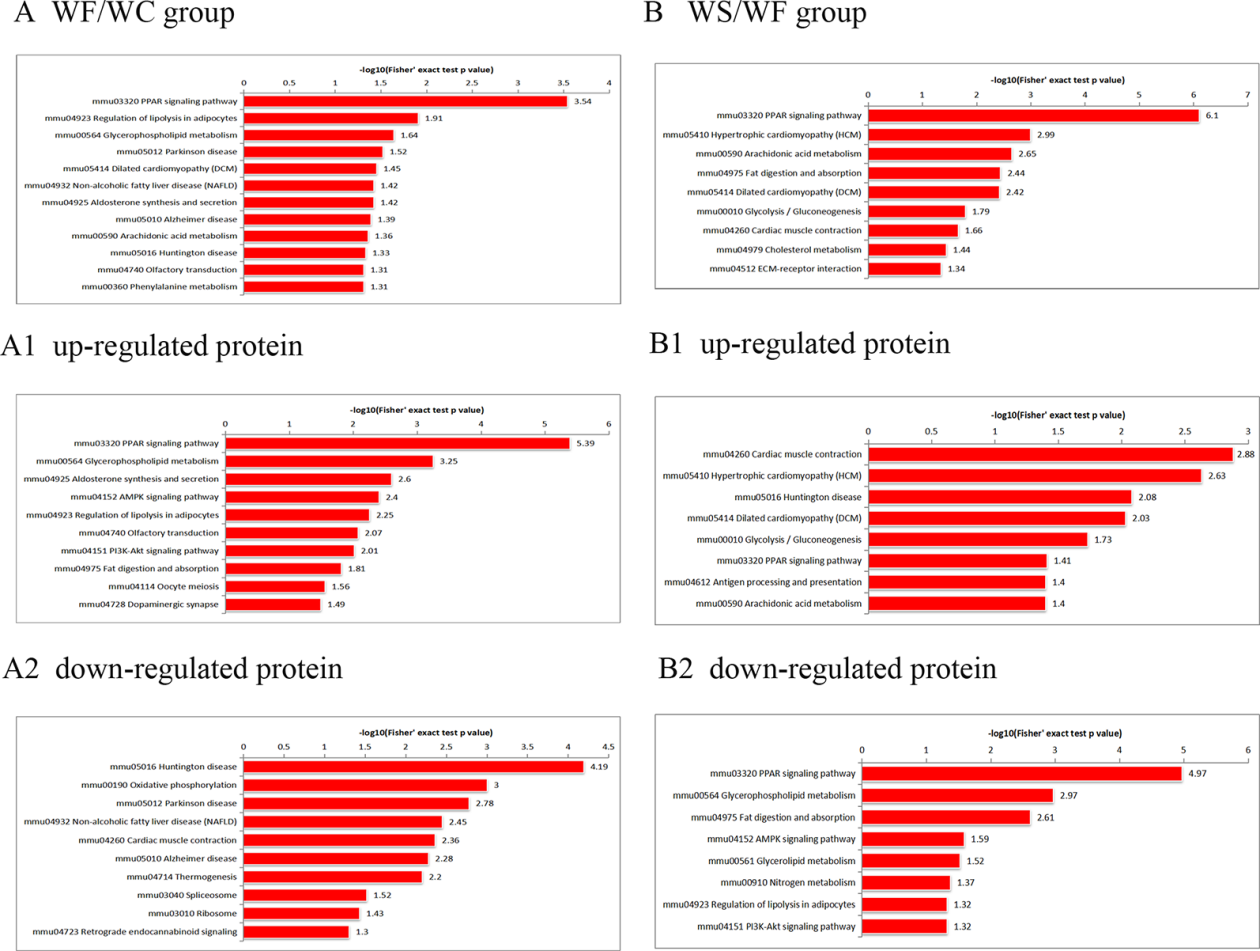
The KEGG pathway enrichment of differentially expressed proteins. **(A)** In WF/WC group, differentially expressed proteins were involved in PPAR, lipolysis of fat cells, metabolism of glycerides, Parkinson's disease, and dilated cardiomyopathy pathways; **(A1)** The pathway of up-regulated proteins; **(A2)** The pathway of down-regulated proteins; **(B)** In WS/WF group, the differential proteins were mainly concentrated in PPAR, hypertrophic cardiomyopathy, arachidonic acid metabolism, fat digestion and absorption, and dilated cardiomyopathy metabolic pathway; **(B1)** The pathway of up-regulated proteins; **(B2)** The pathway of down-regulated proteins; WC, normal diet; WF, high fat diet; WS, high fat diet + silibinin.

**Figure 8:**
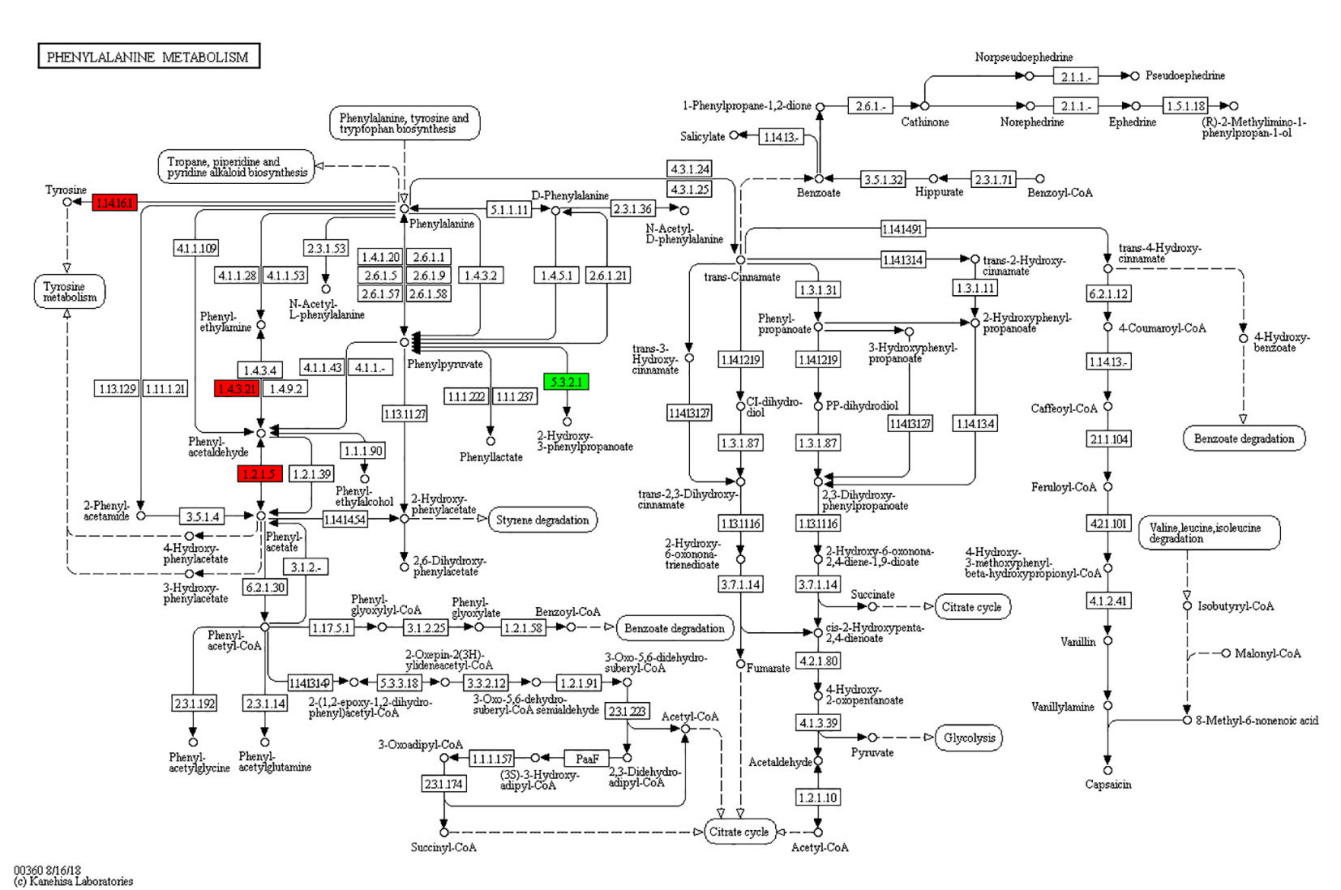
Network of phenylalanine metabolic pathways.

**Figure 9:**
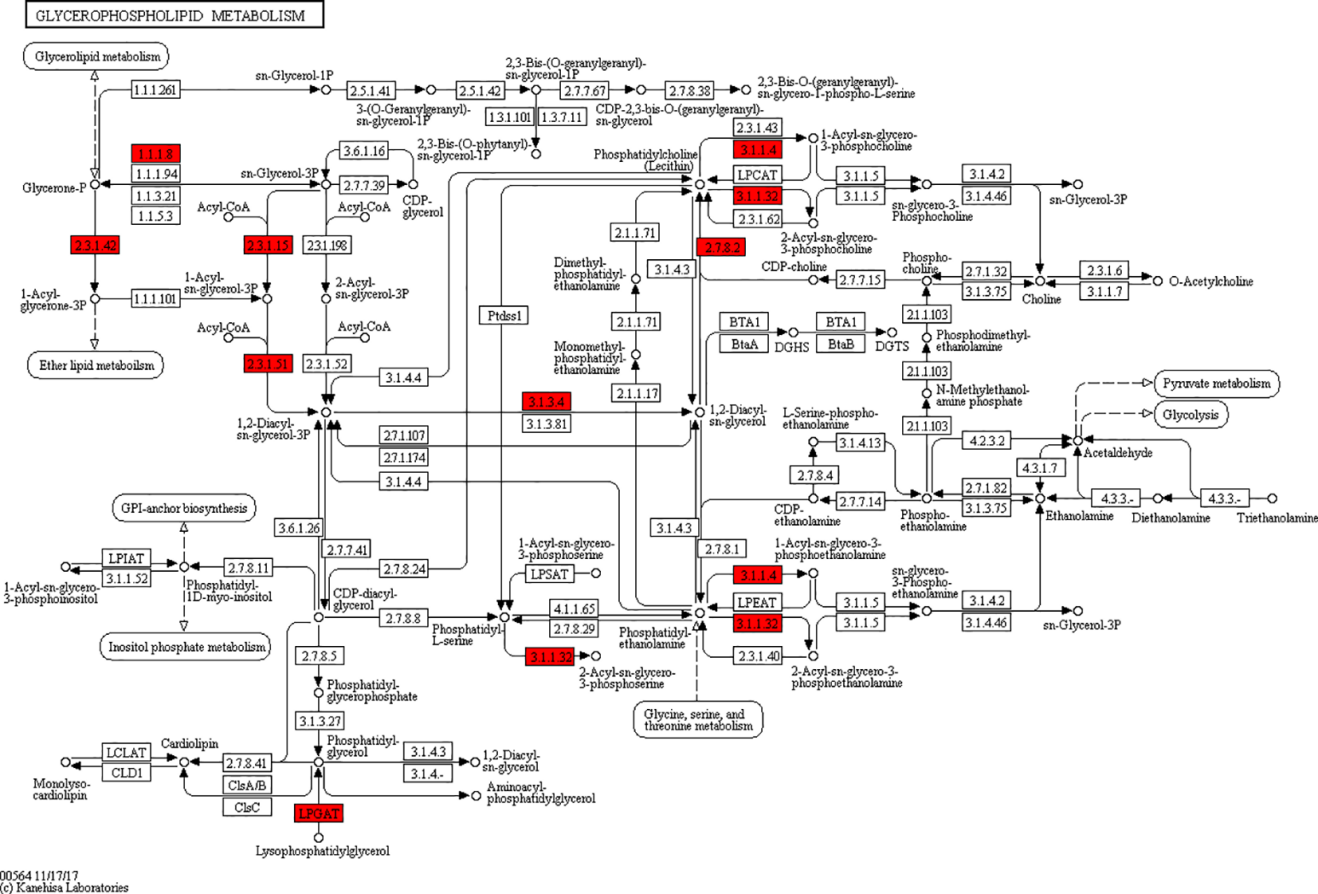
Network of glycerophospholipid metabolic pathways.

**Figure 10:**
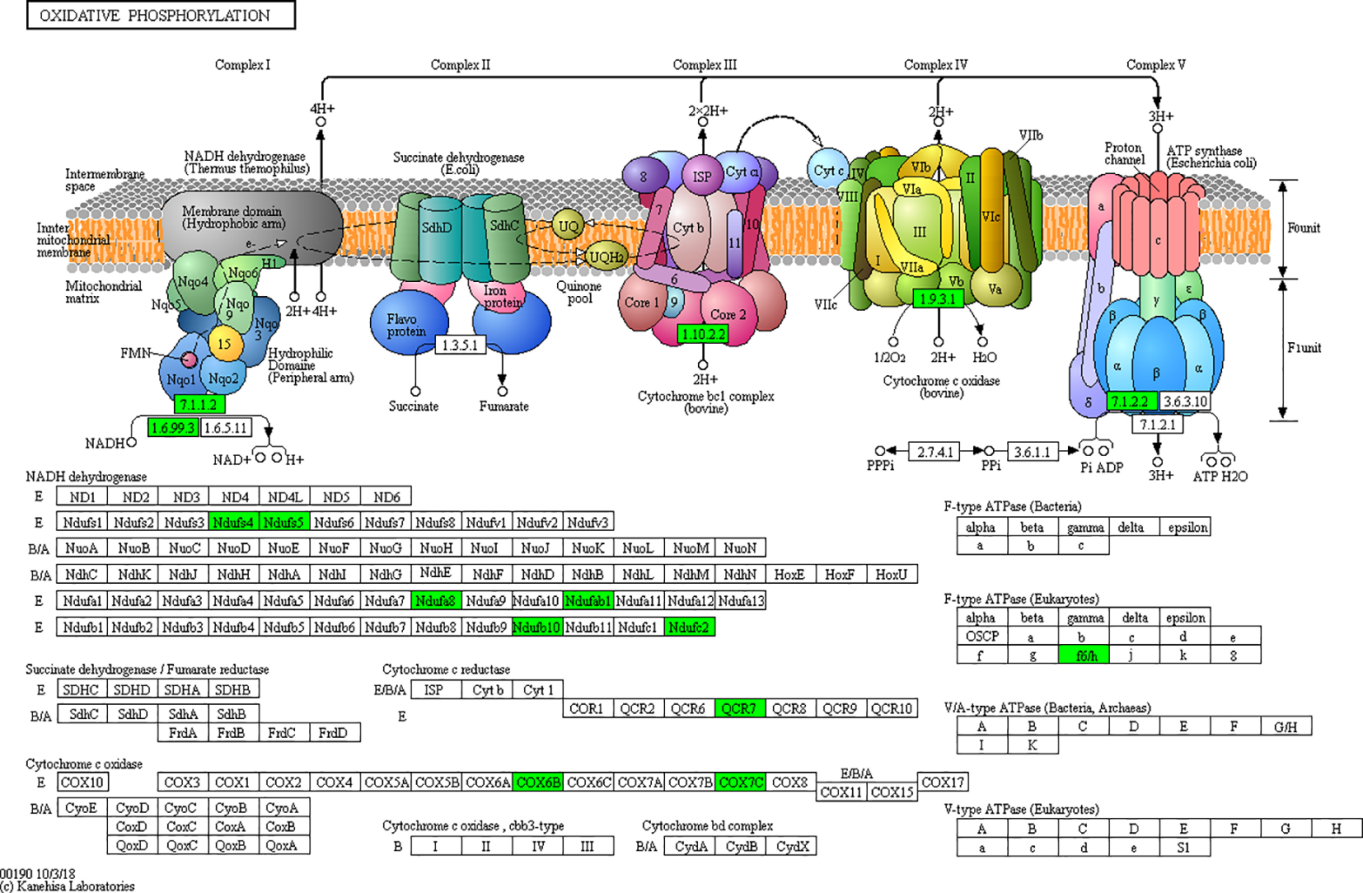
Network of oxidative phosphorylation.

According to the KEGG pathways, the differentially expressed proteins in WF/WC group were involved in PPAR, lipolysis of fat cells, metabolism of glycerides, Parkinson's disease, and dilated cardiomyopathy pathways (**Figure 7A**). Among them, up-regulated proteins are involved in PPAR, metabolism of glycerophospholipids, synthesis and metabolism of aldosterone, and lipolysis in AMPK and adipocytes (**Figure 7A1**). Down-regulated proteins are mainly involved in Huntington's disease, oxidative phosphorylation, Parkinson's disease, non-alcoholic fatty liver disease, and myocardial contractile metabolic pathway (**Figure 7A2**), while in the WS/WF group, the differential proteins were mainly concentrated in PPAR, hypertrophic cardiomyopathy, arachidonic acid metabolism, fat digestion and absorption, and dilated cardiomyopathy metabolic pathway (**Figure 7B**). The up-regulated protein were mainly involved in myocardial contraction, hypertrophic cardiomyopathy, Huntington's disease, dilated cardiomyopathy, and glycolysis process (**Figure 7B1**). Down-regulated proteins are mainly involved in the metabolism of PPAR, glycerides, digestion and absorption of fat, and metabolic processes of AMPK and glycerides (**Figure 7B2**). Further, by integrating the above results, we found that the differential proteins in the two comparison groups were mainly concentrated in the process of lipid metabolism and energy metabolism (**Figures 8**–**10**).

### Silibinin Down-Regulated the mRNA Expression of FABP5, Plin4, GPD1, and AGPTA2

**Figure 11:**
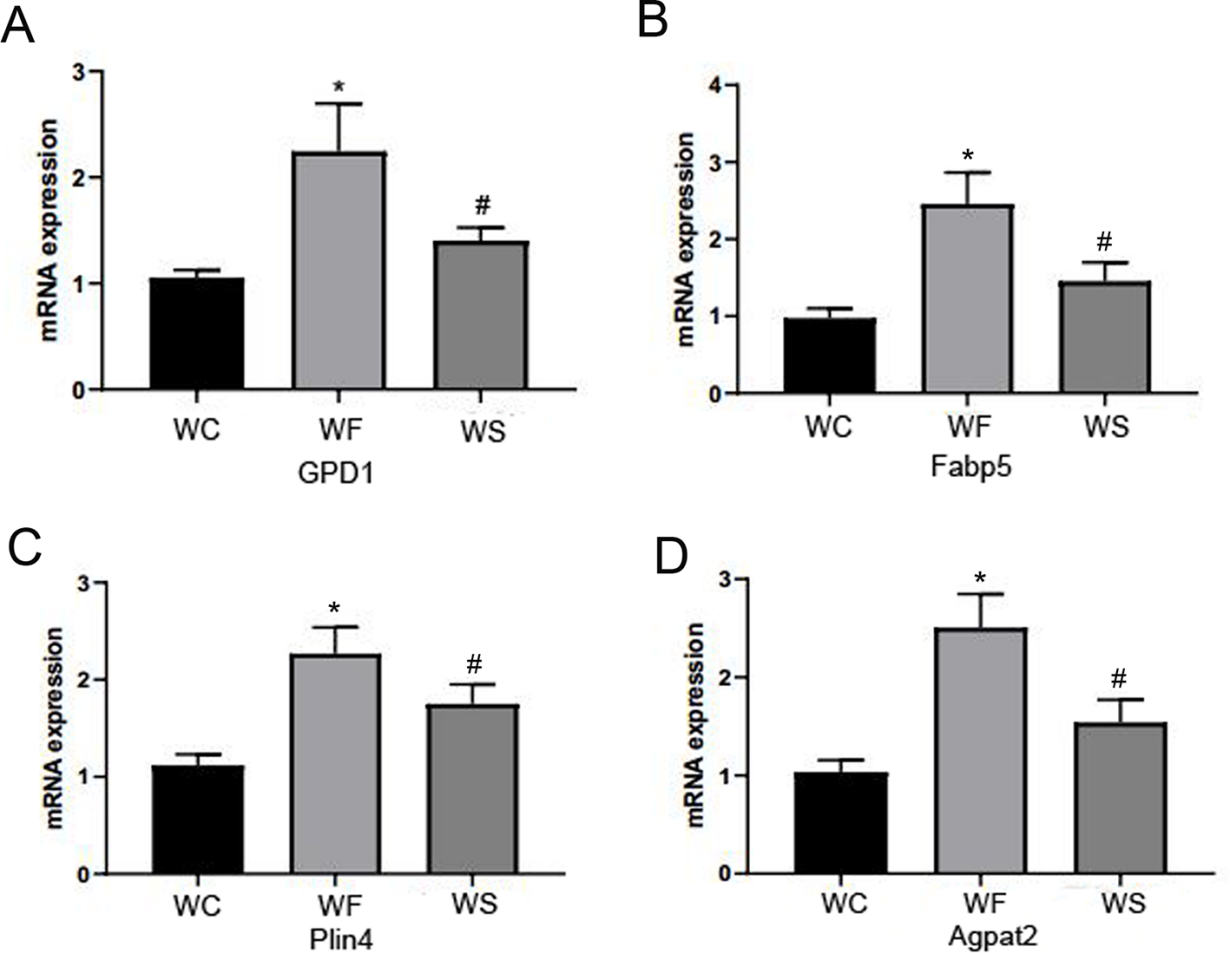
Silibinin down-regulated the mRNA expression of GPD1, Fabp5, Plin4, and Agpta2. **(A)** The mRNA expression of GPD1; **(B)** The mRNA expression of Fabp5; **(C)** The mRNA expression of Plin4; **(D)** The mRNA expression of Agpat2. *P < 0.05 vs WC, ^#^P < 0.05 vs WF.

In **Figure 11**, we observed that compared with the WC group, the expression levels of FABP5, Plin4, GPD1, and AGPTA2 mRNA in the adipose tissue of the epididymis of the WF group were significantly increased, and the differences were statistically significant (*P* < 0.05). Interestingly, the addition of silibinin significantly down-regulated the expression of the four genes in the serum in comparison to the WF group (*P* < 0.05).

### Proteomics Validation

**Figure 12:**
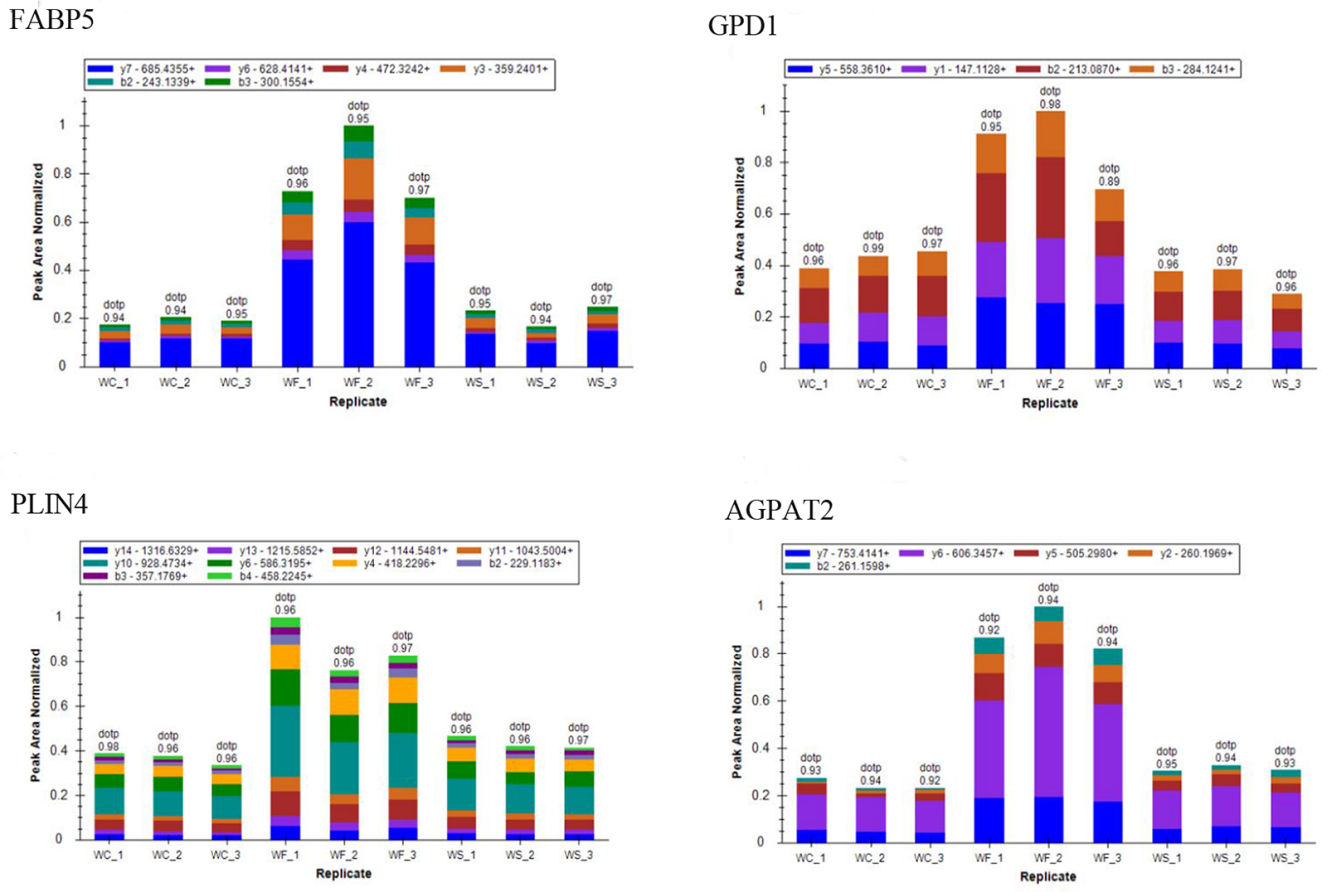
The ion peak area distribution of Fabp5, GPD1, Plin4, and Agpta2 proteins in each sample. After induction of high-fat diet, protein Fabp5, GPD1, Plin4, and Agpta2 increased significantly. When silibinin was added, all the above proteins in WS group were decreased.

To confirm whether differently expressed proteins were consistent with that determined by TMT‐coupled‐LC‐MS/MS, we identified 4 of them. It was found that the expression of fatty acid-binding proteins 5 (Fabp5), glycerol triphosphate dehydrogenase (GPD1), perilipin 4 (PLIN4), and AGPTA2 increased in obese mice induced by high-fat diet, while the expression level decreased after silibinin intervention (**Figure 12**).

## Discussion

A large number of clinical experiments and animal experiments have shown that silibinin has the functions of anti-oxidation, membrane stability, anti-fibrosis, immune regulation, and attenuation of tumor invasion (Tajmohammadi et al., 2018; Suk et al., 2019). In addition, studies have shown that silibinin can regulate the CFLAR-JNK pathway to improve glucose and lipid metabolism, insulin resistance, and oxidative stress in the liver (Thompson et al., 2018). Multiple *in vitro* studies have shown that silibinin can inhibit 3T3-L1 cell adipogenesis, prevent its differentiation, and thus inhibit abiogenesis (Ka et al., 2009). In this study, we used TMT combined with LC-MS/MS to observe the changes of protein levels in WAT of epididymis of obese mice induced by high-fat diet before and after silibinin administration, so as to explore the underlying mechanism of silibinin on WAT. Based on the results of the study, we observed that most of the differential proteins are concentrated in lipid metabolism and energy metabolism. Here, we selected some of them for detailed introduction:

Fatty acid-binding proteins (FABPs) are a kind of lipid chaperone protein that can regulate fatty acid transport, signal transduction, and nuclear transcription (Gan et al., 2015), which is closely related to obesity, type-2 diabetes, cardiovascular disease, tumor, fatty liver, and other diseases (Senolt et al., 2010; Kawaguchi et al., 2016; Thompson et al., 2018). FABPs expression is regulated by various factors such as peroxisome proliferator-activated receptor gamma (PPARγ), free fatty acid (FFA), insulin, and long-chain fatty acids (Liu et al., 2007), which can promote FFA transport to lipid droplets, leading to lipid deposition in adipose tissue (Holmes and Cox, 2012). Meanwhile, it can also increase the transport of FFA to other tissues (liver, skeletal muscle), causing ectopic lipid deposition, which eventually leads to chronic inflammation. By activating PI3K/AKT and MAPK/ERK pathways, FABPs can up-regulate the expression of lipase and fatty acid transporters, down-regulate the expression of lipid-deficient rate-limiting enzymes and fatty acid oxidation-related genes, and aggravate mitochondrial dysfunction, thereby promoting adipocyte proliferation and lipid synthesis and attenuate lipolysis (Ka et al., 2009; Gan et al., 2015; Suh et al., 2015) pointed out that silibinin can regulate cell cycle and down-regulate the expression of lipid-producing genes such as FABP4 and inhibit 3T3l-1 adipocyte differentiation and lipid deposition. However, FABP4 and FABP5 have synergistic effects. Combined with the conclusion of Maeda (Maeda et al., 2005) that the lack of both FABP4 and FABP5 has a stronger protective effect on diet-induced insulin resistance, obesity, and atherosclerosis than any single lack, so we speculated that silibinin may also affect lipid transport through FABP5.

Lipids are one of the body's most important energy sources, and lipid droplets (LDs) are the basic structure of adipose tissue, whose function is related to many protein molecules in cells. Lipid droplets coat proteins (PLINs) family included perilpin, ADRP, and TIP47, then S3-12 and MLDP protein was discovered (Wu et al., 2015). A previous study has indicated that inactivation of PLIN4 down-regulated PLIN5 and reduced cardiac lipid accumulation in mice (Chen et al., 2013). Recently, another study has confirmed that plin2-/- mice can prevent obesity by inhibiting PPARγ, SREBP-1, and SREBP-2 pathways to improve insulin sensitivity, relieve endoplasmic reticulum stress, and inhibit the expression of lipid-synthesis related proteins (Libby et al., 2016).

The glycerophosphate pathway is an important pathway for *de novo* synthesis of triglycerides, and AGPAT2 plays a key role in the synthesis of glycerolipid and triglyceride. As a precursor of *de novo* synthesis of major glycerides (Bradley et al., 2017), it is highly expressed in adipose tissue and liver and can catalyze the formation of triglycerides (Fernandez-Galilea et al., 2015). AGPAT2 mutation can lead to congenital systemic adipotrophic diabetes (GCL), which is characterized by adipose tissue deficiency, insulin resistance, diabetes, and hyperlipidemia (Oswiecimska et al., 2019). AGPAT2 can also regulate adipose tissue by altering the activation of PI3K-AKT and PPARγ (Blanchard et al., 2016), and the accumulation of triglycerides in adipocytes can be reduced by knocking AGPAT2 (Gale et al., 2006). Glycerol triphosphate dehydrogenase (GPD1) is a rate-limiting enzyme (Remize et al., 2001) that catalyzes the synthesis of glycerol, linking carbohydrate and lipid metabolism. It was able to reduce dihydroxypyruvate phosphate (DHAP) to glycero-3-phosphate (G3P), which provided a substrate for the synthesis of triglycerides. Overexpression of GPD in green algae by Wang et al. increased lipid content and triglyceride accumulation in green algae (Wang et al., 2018).

In this study, it was found that the expression of fat transporter FABPS and lipid synthesis protein PLINS, AGPAT2, and GPD1 in the adipose tissue of obese mice increased, while their expression decreased after the intervention of silibinin; further verification results and mRNA expression of FABP5, PLIN4, GPD1, and AGPAT2 were consistent with our proteomics results. In addition, in our study, we also observed that high-fat diet could increase the concentration of TC, TG, LDL-C, and HDL-C in mice, while after silibinin intervention, the concentration of TC, TG, and LDL-C in mice decreased, and the glucose metabolism capacity was significantly improved. More notably, in another set of our studies (the same batch of mice), lipid deposition in the liver was significantly reduced by oil red staining of the liver tissue (unpublished data). Therefore, it can be speculated that high-fat diet can promote the expression of fat transporter and lipid-synthesis related proteins, promote FFA transport and FFA re-esterification, further promote TG deposition in fat cells and ectopic deposition in liver and skeletal muscle, and finally induce obesity and other metabolic syndrome manifestations in the body. On the other hand, silibinin can reduce lipid accumulation and ectopic deposition, improve lipid metabolism in the body, and avoid the occurrence of diseases related to metabolic syndrome such as insulin resistance, cardiovascular disease, type 2 diabetes, and obesity by down-regulating the expression of related proteins.

Mitochondria play an important role in biological processes such as oxidative stress, inflammation, apoptosis, and metabolic function and are closely related to neurodegenerative diseases, obesity, dyslipidemia, cancer, and other diseases (Cherry and Piantadosi, 2015; Woo et al., 2019). This is also consistent with our findings that the down-regulated differential proteins in the control group and the high-fat group were mainly concentrated in the disease pathways such as cardiomyopathy and Alzheimer's disease. Mitochondrial dysfunction in adipose tissue is a key factor of adipose tissue inflammation, which can lead to accumulation of triglycerides, enlargement of fat cells, and hypoxia of fat, which will further aggravate inflammation and mitochondrial dysfunction (Novak, 2012). Oxidative phosphorylation (OXPHOS) is the most important metabolic pathway in mitochondria, maintaining cellular energy homeostasis (Melcher et al., 2017). Mitochondrial CI (NADH), the first enzyme in the mitochondrial electron transporter, is the ATP that drives the mitochondrial ATP synthase. Nadufa8, Nadufs4, Nadufs5, and Nadufc2 all participate in CI assembly and its stability. Ndufs4+/- mice can lead to partial loss of CI activity and stability of mitochondrial complex, resulting in oxidative stress (Alam et al., 2015). Ndufab1-/- down-regulated genes in the ventricle of mice are mainly related to mitochondrial metabolism and mainly involved in metabolic process, fatty acid metabolism, lipid homeostasis, and other processes (Hou et al., 2019). In this study, we observed that mitochondrial related proteins such as Nadufa8, Nadufs4, Nadufs5, and Nadufc2 were down-regulated in obese mice, and their expressions were up-regulated after silibinin intervention. Therefore, we hypothesized that a high-fat diet could induce the down-regulation of mitochondrial protein, causing mitochondrial dysfunction and oxidative stress, leading to an increased risk of fatty tissue inflammation, insulin resistance, fatty liver, neurodegeneration, and other diseases, while the intervention of silibinin can up-regulate mitochondrial protein expression, improve mitochondrial dysfunction, and reduce oxidative stress, adipose tissue function, inflammation, and insulin resistance.

Numerous clinical and demographic studies have shown that elevated serum bilirubin levels can prevent cardiovascular and metabolic diseases such as obesity and diabetes. Bilirubin is an effective antioxidant, and the beneficial effect of appropriately increasing plasma bilirubin is thought to be due to the antioxidant effect of this bile pigment (Stec et al., 2016). However, apart from its function as an antioxidant, other physiological functions of bilirubin have not been well explored (O'Brien et al., 2015), and moreover, the antioxidant function of bilirubin alone does not explain how it transcribes signals. Recent work has shown an unexpected action of bilirubin, where it functions as an agonist for peroxisome proliferator-activated receptor alpha (PPAR-α) (Stec et al., 2016). PPAR-α is a nuclear receptor whose ligand activation binds to promoters of thousands of genes to increase fat burning and reduce fat storage (Montagner et al., 2016). The latest research shows that bilirubin may regulate transcriptional responses through PPAR-α to improve hepatic dysfunction (Hinds et al., 2017). In addition, it was reported that bilirubin treatment may improve HFD-induced insulin resistance by promoting a favorable adipocytokine profile *via* the inhibition of oxidative stress (Takei et al., 2019). UGT1A1 is abundant in liver and responsible for the metabolism of numerous drugs and endogenous substances (e.g. bilirubin) (Ma et al., 2017). In addition to bilirubin, it is also important for a variety of other endogenous substances and foreign organisms, including natural agents often used as nutraceuticals, including the flavonolignans and flavonols of the silymarin complex. Studies have shown that silibinin may inhibit bilirubin glucuronosylateion through UGT1A1 (Sridar et al., 2004; Gufford et al., 2014). More interesting, in a recent study, Gordon et al. (2019) showed that RNA sequencing in human HepG2 hepatocytes reveals that PPAR-α mediates transcriptome responsiveness of bilirubin. In our study results, we also found that up-regulation of differential proteins in the PPAR-α pathway is particularly significant. In addition, a mild uncombined hyperbilirubinemia has been demonstrated in patients treated with high doses of silibinin (Marino et al., 2013). In other studies of chronic hepatitis C patients treated with silibinin, significant increases in serum bilirubin levels have also been reported (Neumann et al., 2010; Beinhardt et al., 2011), that is, intracellular and systemic concentrations of bilirubin increase after silibinin exposure. In the following mechanism study, we will focus on the relationship between silibinin, PPAR-α, and UGT1A1, expecting to find a new target.

There are still some limitations in our manuscript. Firstly, the study lacked the hepatic lipid analyses. This is because another subject of our experimental group is the study of liver tissue, so the part of biochemical data belongs to our unpublished part, which we cannot provide in this study. What we can say is that the liver tissue results also show that differential proteins are closely related to fatty acid metabolism, glucose metabolism, oxidative stress, inflammatory response, and PPAR signaling pathways. Secondly, because our serum has been used up during the experiment, we have not measured some important adipokines and the concentration of silibinin in the serum. Finally, we did not observe weight loss in mice after silibinin intervention, which we hypothesized that the cause of this phenomenon may be related to the time of intervention, drug dosage, and drug utilization (Mendez-Sanchez et al., 2019).

## Conclusion

Silibinin can reduce the expression of lipid synthesis and transport-related proteins in obese mice and increase mitochondrial related proteins, thereby reducing lipid synthesis, increasing energy consumption, and improving lipid metabolism *in vivo*.

## Data Availability Statement

The data can be found in Proteome exchange using the accession number PXD016635.

## Ethics Statement

The animal experimental procedures were approved by the Animal Ethics Committee of Hebei General Hospital.

## Author Contributions

SC and LR conceived and designed the experiments. FW provided materials and samples and analyzed the data. YW, ZL, TS, HZ and QY collected and collated the data. All the authors have approved the manuscript.

## Conflict of Interest

The authors declare that the research was conducted in the absence of any commercial or financial relationships that could be construed as a potential conflict of interest.
